# Influencing Factors on Career Preparation Behavior of Nursing Students in the Post COVID-19 Era

**DOI:** 10.3390/nursrep14010042

**Published:** 2024-03-05

**Authors:** Heejung Choi, Vasuki Rajaguru

**Affiliations:** 1 Department of Nursing, Uiduk University, Gyeongju 38004, Republic of Korea; hjchoi@uu.ac.kr; 2 Department of Healthcare Management, Graduate School of Public Health, Yonsei University, Seoul 03722, Republic of Korea

**Keywords:** pandemic, nursing career, career preparation, nursing students, nursing image, self-efficacy

## Abstract

This study aims to determine the factors influencing the career preparation behavior of nursing students in the post-COVID-19 era and to provide a basis for preparation strategies to enhance nursing students’ nursing professionalism and career preparation behaviors. This is a descriptive cross-sectional study that measures major satisfaction, self-efficacy, nursing image, nursing professionalism, nursing image and intuition, and career preparation to identify factors influencing nursing students’ career preparation behavior in the post-COVID-19 era. An online survey was conducted to collect the data. The data were analyzed using descriptive statistics, Pearson’s correlation, and multiple regression analysis using the SPSS/WIN 25.0 program. A total of 240 students were included; most of them were female (86.3%) and between 21 and 25 years old (80%). The level of motivation to pursue nursing (F = 12.34, *p* < 0.001) and clinical practice satisfaction (F = 11.37, *p* < 0.001) showed statistically significant differences in career preparation behavior. Self-efficacy (r = 0.32), major satisfaction (r = 0.32), nursing image (r = 0.32), and nursing professionalism (r = 0.32) were positively correlated with career preparation behavior and significant (*p* < 0.001). According to the findings, nursing professionalism and image can be enhanced by providing career planning and counseling based on the student’s degree of comprehension and cognitive behaviors to nurture the professional and positive attitudes that are essential for a successful nursing career. Nursing schools need to incorporate a job portal, facilities, and a mentorship program to help nursing students prepare for their careers.

## 1. Introduction

The coronavirus disease (COVID-19) pandemic posed a major threat to human health because of its contagious nature and substantial problems in 2020 [1]. The prolonged existence of the global pandemic has made an impact on several facets of life, including the shortage of nurses as healthcare workers, and reports indicate that extended working hours are increasing fatigue and safety challenges [2]. The COVID-19 pandemic has highlighted the flaws in healthcare systems, which highlights the critical need for all governments to make significant investments in nursing to bring about such profound changes [3].

The global propagation of the pandemic has resulted in a significant impact on mental health [3]. Nurses accounted for the highest proportion of medical workers, at 73.5%, due to the nature of their work of directly caring for patients, and it was stated that one nurse per day is infected with COVID-19 [4]. Pandemic-related negative impacts on nursing students’ mental health include fatigue, stress, and anxiety [5]. Nursing students were reported to have a significant level of risk awareness as well as anxiety [5,6,7]. Thus, there are concerns that it may cause people to be hesitant to seek a career as a nurse owing to the stress and anxiety about work [5,6]. Good nursing professionalism has an impact on effective nursing work performance and low turnover rates [8]. A distinct road map is becoming necessary to provide a clear path for suitable nursing education and differentiated nursing services, making use of this crisis as an opportunity [9]. The COVID-19 pandemic strengthened the professional identities of nursing students, according to a nationwide study conducted in China [10]. Individuals who identified more strongly as professionals planned to stay in nursing and had more optimism during the pandemic. However, failing mental health has been associated with challenges in learning and job preparation attitudes toward nursing as a career. In the COVID-19 crisis, nursing students believe that it is time to redefine the nursing profession [5,10] and have an opportunity to transform their professional identities as well as address healthcare issues with the COVID-19 pandemic [6,11].

Currently, nursing image is changing positively, but nursing still has negative social images, such as being difficult jobs, having a rigid organizational culture, and being feminine jobs [12,13,14]. Nursing plays a significant role in public health according to a systematic review that investigated society’s opinions about the nursing profession [15]. Conversely, it has been disclosed that nurses are seen as subordinates to physicians, carrying out medical treatments as instructed by physicians, complying with physicians’ orders without question, and enduring extended work hours and meager salaries [16,17]. Societies’ images of nursing after the pandemic were unique from their images of nursing before the pandemic [6,18]. This negative image of nursing is an obstacle to choosing nursing as a career, but a positive image of nursing gives value to one’s work and makes one feel proud [18]. Therefore, by examining the relationship between nursing students’ career preparation behavior and nursing image, it is necessary to improve career preparation behavior by forming a correct nursing image during college and to help them adapt to the job later.

Major satisfaction is the result of satisfying one’s needs while studying one’s major while also combining positive thoughts about enjoyment, one’s future, and a career path [19,20,21,22]. The nursing curriculum included more elective subjects that were directly relevant to the major and was quite stressful because of theory and clinical practice, which lowers major satisfaction [22]. In addition, many students enroll in the nursing department because of its high employment rate, which can lead to dissatisfaction with the major because their aptitude does not match the major after admission [23]. A number of factors have been found to have an impact on overall student satisfaction in higher education, including the effectiveness of the instruction, the availability of the internet and libraries, the efficiency of the administrative staff, the atmosphere of the university, and student characteristics like age, gender, and ethnicity [24]. In addition, empirical analysis, and a breakdown of the concept of “student satisfaction” indicate that the academic and educational qualities of instruction are significant factors that influence students’ major satisfaction [5,7,10]. These factors may even correlate with the opinions of the instructors themselves. Therefore, it is necessary to understand the impact of nursing students’ level of satisfaction with their major on their career preparation behavior.

Developing self-efficacy may promote confidence and independence [25]. Research indicates that having a high sense of the practice of self-efficacy increases one’s intention to continue in a profession and increases satisfaction with one’s job [9]. Most prior research on senior nursing students had small sample sizes and utilized qualitative approaches to reflect on students’ career preparation and the job-finding obstacles that they faced due to a lack of self-efficacy [13,26,27,28,29]. In addition, few studies have examined students’ prospects or careers after they graduate [21,24,26]. Therefore, it is pertinent to ascertain the correlations between these variables in the context of nursing students nearing graduation amidst the COVID-19 pandemic [17,24]. A new generation of people has experienced the cessation of their clinical practice as a result of COVID-19, and they will be graduating nursing in a contemporary context [27]. It is imperative to comprehend the task-performing capabilities of the emerging group of nurses, considering their distinct experiences in virtual practice and innovative educational programs that diverge from those of the current nursing profession.

Career preparation behavior is a practical and concrete activity that involves making behavioral efforts through various methods to make the right career decision [30,31]. The COVID-19 pandemic has not only disturbed academic activities, but also had a significant impact on students’ employment preparation activities, such as a lack of clinical practice, lack of communication between mentors and teachers, stress, and a lack of internship opportunities. These resulted in the loss of career information opportunities as well as uncertainty while making career decisions [17]. Moreover, hospitals stopped providing career information workshops and continuing simulation training that offer comprehensive information about their procedures through internships and briefing sessions, which are valuable resources for those making career decisions [15,17,22]. As a result, students worry that they will not have enough real-world experience, which could impact their employment prospects. However, many hospitals chose to cancel or reduce the hosting of such events due to their policies on accepting COVID-19 patients and worries about the spread of infection. There are several factors that affect nursing students’ career preparation behavior in the post-COVID-19 era [17,18]. Study results indicate that 90% percent of college students experienced the repercussions of course suspension. Consequently, their professional and practical coursework was limited thus lowering their competitiveness in the employment market [14,16,17].

Many students became highly afraid and maladjusted after the COVID-19 pandemic and prone to negative emotions and psychological problems like tension, anxiety, irritability, stress, helplessness, and depression because of multiple pressures like patient approach, shift-working, home or remote services, and preventing pandemics, in addition to abrupt changes in the pace of life. This lead to a sharp decline in nursing students’ psychological well-being. Research indicates that those with greater employment adaptability can withstand transitions in their careers more easily and improve wellbeing, even when pandemics are unpredictable and impossible to manage. These factors can be categorized into personal, educational, institutional, and environmental aspects. It’s important to note that the impact of these factors may vary among individuals and across different contexts. To this end, it is necessary to identify various factors that influence the career preparation behavior of nursing students.

The purpose of this study was to identify the significant relationship between career preparation behavior and related factors among nursing students in the post COVID-19 era, as well as the factors that provide the basis for developing strategies to improve nursing students’ professionalism and career preparation behaviors.

## 2. Methods

This study used a descriptive cross-sectional design using a self-reported questionnaire to investigate and determine the career preparation behavior of fourth grade nursing students.

### 2.1. Setting and Samples

The study’s target population comprised fourth grade nursing students who were enrolled at the ‘U’ University, located in the southern region of the Republic of Korea, and were in the process of job preparation during the study period. Students who did not agree with the study and were not actively enrolled in the nursing program were excluded.

The sample size was computed using the G power 3.1.9 tool (Heinrich-Heine-Universität, Düsseldorf, Germany); the required number of samples was determined at the 0.05 level of significance, a power of 0.90, an effect size of 0.15, and about 358 samples were allowed as per sampling power. A total of 250 students answered the survey; ineligible responses were eliminated, and 240 responses were selected for the final analysis.

### 2.2. Measures

The general characteristics of the nursing students were age, sex, motivation to pursue nursing, clinical practice satisfaction, desired first career path and expected duration of service. Five tools were used, which were originally developed or modified by the Korean authors including: major satisfaction (18 items), self-efficacy (23 items), nursing image (29 items, nursing professionalism (18 questions), and career preparation behavior tools (25 questions) and the details are described below.

### 2.3. Major Satisfaction

This instrument measures academic satisfaction as a key factor in students’ decision-making, which subsequently influences how well they integrate into the academic activities and their performance. This tool was initially constructed by Lee comprising 34 items and modified by Ha [32] and included 18 questions. It consists of four sub-areas: general satisfaction (6 questions), perceived satisfaction (6 questions), subject satisfaction (3 questions), and satisfaction with the relationship between professors and students (3 questions). It is measured on a 5-point Likert scale, with higher scores indicating higher major satisfaction. The reliability of the original tool, Cronbach’s alpha, was 0.92, and it was 0.92 in the present study.

### 2.4. Self-Efficacy

The general self-efficacy scale (GSES) was employed in this study to measure self-efficacy. It is a tool used to estimate an individual’s ability to adjust to everyday problems after exposure to a stressful circumstance such as COVID-19. It was developed by Sherer et al. [33] and translated by Hong [34]. The tool consists of 23 items and each question asked the students to rate their answers on a five-point Likert scale ranging from “always” (5) to “never” (1). Good self-efficacy was reflected by higher overall scores. Cronbach’s alpha for the Korean version was 0.89, while it was 0.87 in the current study.

### 2.5. Nursing Image

The nursing image tool is designed to assess the current perception of the brand image of nursing education, practice, work circumstances, and public status. It was developed by Lee et al. [35] and modified and supplemented by Yang [36]. This tool has a total of 29 questions and is composed of 4 subscales: qualifications (9 items), role (7 items), social participation (7 items), and interpersonal relationships (6 items). It is measured on a 5-point Likert scale, and a higher score means a more positive nursing image. The reliability of the original tool, Cronbach’s alpha, was 0.89, and it was 0.91 in the present study.

### 2.6. Nursing Professionalism

This instrument measures a nurse’s clinical efficacy in various domains, such as client care, integrity, communication, conduct, and respect. It was developed by Yeun et al. [37] with 29 questions and was modified and reduced to 18 questions. The tool has been subdivided into 5 sub-scales: professional self-concept (6 items), social awareness (5 items), professionalism in nursing (3 items), role of the nursing community (2 items), and independence of nursing (2 items). It is measured on a 5-point Likert scale, and higher scores indicate higher nursing professionalism. The reliability of the tool, Cronbach’s alpha, was 0.91, and it was 0.85 in the present study.

### 2.7. Career Preparation Behavior

This instrument assesses the career preparation behavior of students that corresponds with their preferences, decision-making, and career identity. This tool was developed by Kim et al. [38]. This tool consists of a total of 25 questions and is composed of three sub-domains: self-understanding behavior (10 questions), vocational ability improvement behavior (9 questions), and occupational world exploration behavior (6 questions). It is measured on a 5-point Likert scale, with a score range of 1 to 5, varying from “very much” (5) to “not at all” (1). The higher the score, the better the career-preparation behavior. The reliability of the original tool, Cronbach’s alpha, was 0.96, and it was 0.93 in the present study.

## 3. Data Collection

The students that qualified to participate in this study were chosen randomly by the researcher. Because of the COVID-19 outbreak, the students received an indirect online message inviting them to participate in the study. The designated survey was distributed and gathered via an online platform. There was no direct interaction between the students and the researcher. Data was collected from January to February 2022. In appreciation of their voluntary participation, all students who completed the survey received a beverage coupon. Prior to completing the self-reported questionnaire, all participants provided written consent, and the entire process took around 40 min. All completed surveys were evaluated as soon as they were received, and participants were asked to submit more information if necessary.

## 4. Data Analysis

Descriptive statistics were calculated to assess the general characteristics of the nursing students and reported as the frequency, percentage, mean, and standard deviation. We applied Cronbach’s alpha coefficient to verify the reliability of the study tool. We performed an independent *t*-test, one-way ANOVA, and Scheffé test post hoc test to examine the career preparation-related behavior according to the general characteristics of the nursing students. The correlation between major satisfaction, self-efficacy, nursing image, nursing professionalism, and career preparation behavior was analyzed using Pearson’s correlation. Multiple regression analysis was utilized to find the associated factors influencing career preparation behavior and selected variables (grouped into satisfied and not satisfied). All the data were analyzed using SPSS 25.0 for Win (BM Corp., Armonk, NY, USA), and the statistical significance level was considered at the *p* < 0.05 level.

## 5. Ethical Considerations

The study was approved by the Institutional Review Board of ‘U’ University (IRB No. 1041553-202203-001-02). The study’s purpose and procedures were explained, and informed consent was obtained from the participants prior to data collection. In addition, the participants were provided with information regarding their voluntary status and were granted the freedom to withdraw from the study at any time.

## 6. Results

### General Characteristics of the Participants

**Figure 1:**
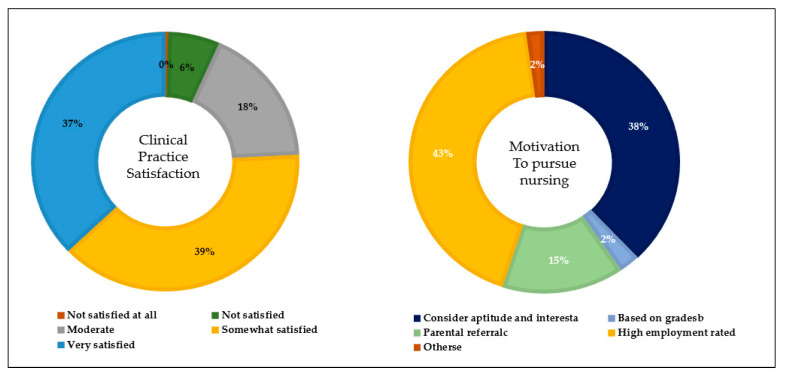
Percentage distribution of clinical practice satisfaction and motivation to pursue nursing among nursing students.

Table 1 presents the general characteristics of the nursing students. Most of the students were female (207, 86.3%), 21 to 25 years old (192, 80%). Most of the students showed the motivation to pursue nursing due to its high employment rate (103, 42.9%) and were somewhat satisfied (93, 38.7%) in their clinical satisfaction. Most of them showed that their first desired job was to work at a hospital (96.7%) and showed a duration of service of about 1 to 3 years (107, 44.6%). The percentage distribution of clinical practice satisfaction and motivation to pursue nursing among nursing students is presented in Figure 1. The level of motivation to pursue nursing (F = 12.34, *p* < 0.001) and clinical practice satisfaction (F = 11.37, *p* < 0.001) showed statistically significant differences in career preparation behavior.

Table 2 illustrates the correlation between self-efficacy, major satisfaction, nursing image, nursing professionalism, career preparation behavior. Self-efficacy (r = 0.51), major satisfaction (r = 0.52), nursing image (r = 0.36), and nursing professionalism (r = 0.43) were positively correlated with career preparation behavior and showed statistically significant (*p* < 0.001).

Table 3 shows the subgroup analysis of the factors influencing career preparation behavior among fourth grade nursing students. Multiple regression analysis findings of the nursing students, scored as a selected dependent variable, exhibited that motivation to pursuing nursing (β = 1.69, t = 0.29, *p* < 0.001), self-efficacy (β = 0.83, t = 5.57, *p* < 0.001) and major satisfaction (β = 0.28, t = 2.52, *p* < 0.05) were found to be statistically significant in career preparation behavior. However, nursing image and clinical practice satisfaction did not show a relationship with career preparation behavior and were not significant. According to the goodness-of-fit test, the regression model was adequate (F = 16.04, *p* < 0.001) with an explanatory power of around 46%.

## 7. Discussion

This study aimed to identify the extent of nursing professionalism among nursing students in the post-COVID-19 era and to identify the influencing factors of career preparation behaviors. Most of the study’s participants were female and 21 to 25 years old. It was found that students were motivated to pursue nursing due to its high employment rate [11,13]. Similar results were reported among participants who were women, younger than 22 years old, and had less than one year of professional experience [18]. More than half of them intended to work as nurses for about 1–3 years in a hospital. These results suggest that nurses have a lower desire to become nurses than do undergraduate nursing students. All nurses who were caring for COVID-19 patients now felt more like professionals, but their desire to become nurses decreased because of their fear of infection, their experience of burnout from a challenging workload, and the way their daily lives and those of their families were getting worse [7,8,9,13]. This was reinforced by undergraduate nursing students with COVID-19 field experience having a low nursing intention toward COVID-19 patients in addition, to determining the relationship between nursing image, nursing professionalism, self-efficacy, and satisfaction with the major with career preparation behavior.

The difference between the career preparation behavior based on the general characteristics showed significant differences in the motivation to pursue nursing and clinical practice satisfaction [16]. Unexpected and abrupt changes in learning methods without the necessary infrastructure and learning systems may be less effective, even though online learning is said to be helpful in nursing education [16]. The lack of clinical experience had a significant impact on preparation for employment [24]. Prior studies reported that students who showed high satisfaction with clinical practice had high nursing professional value [14,15,18] and other studies also documented associations between these factors [3,11,14,15,16,18,21]. In contrast, prior studies have reported that the more career preparation behaviors are performed, the higher the job satisfaction [13,14], and it was found that the likelihood of changing jobs increases [15,16,17]. All these factors could be utilized to improve career preparation behavior and create an efficient career development strategy. Consequently, it is necessary to pay attention to the mental health of nursing students and offer them psychological support while they prepare for their careers. In addition to job counseling and psychosocial support, an on-campus counsellor might carry out routine mental wellness screenings for students.

According to this study, self-efficacy was correlated with the career preparation behavior after COVID-19. This finding is consistent with prior research that demonstrated a positive correlation with the level of contentment. Previous studies of nursing students extensively reported that self-efficacy and coping strategies highly impacted job preparation and behavioral changes [9,33,34]. Norwegian baccalaureate nursing students’ satisfaction with their educational curriculum and overall self-efficacy were studied [26]. More precisely, it was noted that nursing student’s general quality of life and well-being during the COVID-19 pandemic were linked to their level of satisfaction with their nursing major. However, professor–student relationships become more challenging for nursing students to navigate in terms of seeking advice and effective communication for career preparation in the post-COVID-19 era [22]. Furthermore, senior students’ sense of social duty increases with their level of self-efficacy and satisfaction with their nursing major.

Our findings revealed that nursing image and nursing professionalism were positively associated with career preparation behavior. This result was consistent and nursing students had high nursing professional values before clinical practice, but then they were influenced by negative factors from witnessing and experiencing the performance of their duties and the roles of nurses in hospitals in practice [26,27,28]. Major emergencies affect the perspective of students on the meaning of work and life, thereby damaging the impulse to seek out meaningful pursuits [26]. A previous study revealed that professionalism was influenced by self-efficacy [9,21,27,39]. In addition, self-efficacy plays an important role in professionalism [28] and it was found that as the strongest predictor, it also influenced the career preparation of nursing students [30]. The COVID-19 pandemic highlighted the flaws in healthcare systems, which highlights the critical need for all governments to make significant investments in nursing in order to bring about such profound changes [16,18]. On the contrary, the negative impact is more likely to be the reason that students quit the nursing program rather than a lack of competence. Therefore, educational programs and coping strategies are required to promote student retention and enhance their sense of professional identity and professionalism.

Our findings showed that even though these nursing students thought that they were proficient in some areas such as emergency, intensive care units, etc., they should be prepared using additional online or offline training or practice during the job preparation period in specific areas of specialization to feel more prepared for the job. Nursing institutions and policy makers must plan for career coping continuing education programs, including counselling centers, the availability of job portals and workshops, or alumni meetings, to motivate them to obtain additional guidance while in preparation for their employment. On the contrary, education institutions could provide a variety of information regarding career choices using online resources. As a result, guidance and knowledge of the students’ surroundings are beneficial. It would be beneficial to assist students in exploring career options by providing them with correct information and putting them in touch with organizations that offer online assistance with job searching.

There are several limitations to this study. First, all the participants in the study were from a single university. The generalizability of our findings may be limited due to difficulties in confirming causal effects. Second, an online survey was used for this cross-sectional investigation because of the COVID-19 pandemic. It is suggested that longitudinal studies be carried out in the future to evaluate cognitively based career preparation behavior in response to finding the relationship between psychological challenges during career preparation and nursing image changes after COVID-19. Lastly, it solely included fourth grade students; third grade students were not included. Future research should include these students to acquire reliable findings and promote awareness of early career preparation behaviors.

## 8. Conclusions

This study found that the majority of fourth grade students reported the motivation to pursue nursing due to its high employment rate. In contrast, they expressed great satisfaction and willingness to work as nurses in a hospital. However, the expected length of employment was one to three years. This suggests that there may be uncertainty surrounding the professionalism and the image of nursing. Furthermore, career planning behavior was strongly connected to major satisfaction, nursing image, nursing professionalism, and self-efficacy. Understanding and addressing these influencing factors can help educational institutions, policymakers, and healthcare organizations better support nursing students in their career preparation journey in the post-COVID-19 era. Nursing educators are recommended to enhance nursing students’ professionalism, career preparation, and education, and counselling programs that consider their academic year must be developed. In addition, it is essential to establish many distinct professional preparation environments and job portals, as well as to design a hybrid-type career mentoring program and counseling that can be carried out more objectively within the nursing institution.

## Figures and Tables

**Table 1:** General characteristics of the participants. (N = 240).

Characteristics	Categories	n (%)	Career Preparation Behavior		
			M (SD)	F or t (*p*) *	
Gender	Male	33 (13.8)	89.33 (20.00)	−1.96	
	Female	207 (86.2)	95.41 (15.97)	(0.052)	
Age	21~25	192 (80.0)	94.40 (16.15)	1.17 (0.324)	
	26~30	22 (9.1)	90.23 (17.77)		
	31~35	11 (4.6)	100.64 (17.29)		
	36~40	4 (1.6)	98.50 (17.79)		
	41~45	3 (1.3)	91.33 (13.58)		
	46~50	5 (2.1)	95.00 (30.36)		
	≥51	3 (1.3)	113.00 (5.20)		
Motivation to pursue nursing	Consider aptitude and interesta	91 (37.9)	102.24 (15.36)	12.34 (<0.001)	b > c, d > a, e *
	Based on gradesb	6 (2.5)	71.50 (24.84)		
	Parental referralc	35 (14.6)	89.09 (14.90)		
	High employment rated	103 (42.9)	90.62 (14.90)		
	Otherse	5 (2.1)	102.60 (9.40)		
Clinical Practice Satisfaction	Not satisfied at all	1 (0.4)	-	11.37 (<0.001)	
	Not satisfied	15 (6.3)	83.47 (12.70)		
	Moderate	42 (17.5)	86.10 (18.04)		
	Somewhat satisfied	93 (38.7)	92.61 (13.58)		
	Very satisfied	89 (37.1)	102.45 (16.20)		
Desired first career path	Hospitals	232 (96.6)	94.94 (16.49)	1.68 (0.190)	
	Government employees	3 (1.3)	82.00 (25.51)		
	Other	5 (2.1)	85.400 (18.72)		
Expected duration of service	1~3	107 (44.6)	92.37 (17.45)	1.29 (0.275)	
	4~6	61 (25.4)	98.25 (14.52)		
	7~9	16 (6.7)	94.75 (18.19)		
	10~20	11 (4.5)	97.18 (18.87)		
	≥20	45 (18.8)	94.13 (16.67)		

* Scheffé test post hoc test; M (SD), Mean (Standard deviation).

**Table 2:** Correlation between self-efficacy, major satisfaction, nursing image, nursing professionalism, career preparation behavior (N = 240).

Variable	M (SD)	1 r (*p*)	2 r (*p*)	3 r (*p*)	4 r (*p*)	5 r (*p*)
1. Self-efficacy	36.15 (6.32)	1				
2. Major satisfaction	75.69 (10.02)	0.43 (<0.001)	1			
3. Nursing image	202.96 (21.81)	0.29 (<0.001)	0.48 (<0.001)	1		
4. Nursing Professionalism	73.48 (9.33)	−0.32 (<0.001)	−0.48 (<0.001)	−0.69 (<0.001)	1	
5. Career Preparation Behavior	94.58 (16.67)	0.51 (<0.001)	0.52 (<0.001)	0.36 (<0.001)	0.43 (<0.001)	1

M (SD), Mean (standard deviation).

**Table 3:** Factors influencing career preparation behavior among fourth grade nursing students.

Variable	B *	SE	β	t (*p*)
Motivation to pursue nursing ^a^ (Consider aptitude and interest)	1.69	5.90	0.05	0.29 (<0.001)
Based on grades	−29.42	8.12	−0.28	−3.62 (0.775)
Parental referral	−6.77	6.13	−0.14	−1.11 (0.270)
High employment rate	−6.75	5.86	−0.20	−1.15 (0.250)
Clinical practice satisfaction ^b^ (Not satisfied)	−33.90	14.40	−0.49	−2.36 (0.119)
(Moderate)	−32.12	14.03	−0.73	−2.29 (0.323)
(Somewhat satisfied)	−30.05	13.87	−0.88	−2.17 (0.231)
(Very satisfied)	−27.87	13.90	−0.81	−2.01 (0.246)
Self-efficacy ^‡^	0.83	0.15	0.32	5.57 (<0.001)
Major satisfaction ^‡^	0.28	0.11	0.17	2.52 (0.012)
Nurse image ^‡^	−0.04	0.06	−0.06	−0.78 (0.438)
Nursing Professionalism ^‡^	0.34	0.13	0.19	2.70 (0.007)
		Adj R^2^		0.43
		F (*p*)		16.04 (<0.001)

* Adjusted with age, gender; a = dummy coded (other = 1), b = dummy coded (not satisfied = 1); ‡ = grouped in to two (not satisfied = 1, satisfied = 0).

## Data Availability

All data generated analyzed during the current study are available from the corresponding author on reasonable request.

## Public Involvement Statement

No public involvement in any aspect of this research.

## Guidelines and Standards Statement

This manuscript was drafted against the Strengthening the Reporting of Observational Studies in Epidemiology (STROBE) guidelines for reporting observational studies.
